# Distinct patterns of cell adhesion, migration, and morphology in olfactory neuroepithelium cells of bipolar disorder patients

**DOI:** 10.1186/s10020-024-01039-8

**Published:** 2024-12-23

**Authors:** Alejandra Delgado-Sequera, Jose I. Pérez-Revuelta, Andres Caballero-García, MªCarmen Durán-Ruiz, Cristina Romero-Lopez-Alberca, Clara García-Mompó, Francisco González-Saiz, Manuel Rodríguez-Iglesias, Daniel Sanchez-Morillo, Patricia Robledo, Victor Perez, Esther Berrocoso, Maria Hidalgo-Figueroa

**Affiliations:** 1 Neuropsychopharmacology and Psychobiology Research Group, University of Cadiz, Cádiz, Spain; 2 Biomedical Research and Innovation Institute of Cadiz (INiBICA), Cádiz, Spain; 3 Department of Mental Health, Jerez de la Frontera University Hospital, Cádiz, Spain; 4 Severe Mental Disorder Research Group, Department of Neuroscience, University of Cadiz, Cádiz, Spain; 5 Centre for Biomedical Research in Mental Health (CIBERSAM), Instituto de Salud Carlos III, Madrid, Spain; 6 Department of Otolaryngology, Puerta del Mar University Hospital, Cádiz, Spain; 7 Biomedicine, Biotechnology and Public Health Department, University of Cadiz, Cádiz, Spain; 8 Department of Psychology, University of Cadiz, 11510 Cádiz, Spain; 9 Department of Microbiology, Puerta del Mar University Hospital, Cádiz, Spain; 10 Bioengineering, Automation, and Robotics Research Group, Department of Automation Engineering, Electronics and Computer Architecture and Networks, University of Cadiz, Cádiz, Spain; 11 Integrative Pharmacology and Systems Neuroscience Group, Hospital del Mar Research Institute, Barcelona, Spain; 12 Mental Health Research Group, Hospital del Mar Medical Research Institute, Barcelona, Spain; 13 Department of Neuroscience, University of Cadiz, 11003 Cadiz, Spain

**Keywords:** Bipolar disorder, Cytoskeleton, Biomarker, Olfactory neuroepithelium

## Abstract

**Background:**

Bipolar disorder (BD) is a severe, chronic mental illness that remains difficult to diagnose due to the lack of specific biomarkers, relying primarily on clinical assessments. Early diagnosis and treatment are essential for improving prognosis and lowering suicide risk. This study aimed to identify biomarkers and therapeutic targets by utilizing olfactory neuroepithelium (ONE) cells from patients with BD and controls.

**Methods:**

Immunofluorescence of ONE cells, along with proteomic and RNA sequencing analyses, was performed to investigate cytoskeletal changes and pathways involved in cell adhesion, movement, and morphology. Additionally, potential biomarkers were investigated in blood samples to improve clinical accessibility.

**Results:**

Thus, according to functional assays, ONE cells derived from BD patients exhibited decreased substrate adhesion, reduced cell migration, and morphological changes compared to control cells. In addition, proteomic and RNAseq analyses in ONE cells and peripheral blood mononuclear cells (PBMCs) revealed alterations in pathways such as RhoA/PAK/Integrin and Actin Cytoskeleton Signaling, as well as significant changes in inflammatory and immunological pathways. AUROC analysis identified proteins like PTK2 as potential diagnostic biomarkers, showing altered expression in both ONE cells and PBMCs. *PTK2* RNA expression correlated with distinct morphological traits in BD ONE cells.

**Conclusions:**

In summary, this study identified cytoskeletal alterations, reduced adhesion, and disrupted migration patterns in BD ONE cells, highlighting molecular mechanisms underlying these changes and emphasizing PTK2’s role as a potential diagnostic biomarker for BD.

**Supplementary Information:**

The online version contains supplementary material available at 10.1186/s10020-024-01039-8.

## Background

Bipolar disorder (BD) is a severe, chronic mental illness with a global lifetime prevalence exceeding 1%, marked by alternating episodes of mania and depression (Grande et al. 2016). The disorder follows a variable course and is often associated with cognitive impairment, reduced quality of life, and a significantly increased risk of suicide (Gonda et al. 2012; Oldis et al. 2016). Early diagnosis and treatment are essential for improving prognosis and lowering suicide risk. However, diagnosing BD remains challenging due to the lack of specific biomarkers, relying solely on longitudinal clinical assessments (Vieta et al. 2018). Therefore, there is a critical need for novel biomarkers to enhance diagnostic accuracy and guide early, personalized interventions.

BD is a complex disorder influenced by genetic and environmental factors (Vieta et al. 2018). Epidemiological studies have associated these factors with observable abnormalities in patients, such as cerebral defects and altered expression of genes linked to neural progenitor proliferation, migration, and differentiation into mature neurons. Cytoskeletal dynamics play a crucial role in these processes, both during embryonic development and in the maintenance of the adult nervous system (Benítez-King et al. 2016; Yang et al. 2010). Indeed, postmortem analyses of BD patient brains reveal reduced dendritic spine density and synaptic contacts, which correlate with disrupted cytoskeletal protein expression (English et al. 2009; Pennington et al. 2008). Recent studies underscore the importance of cytoskeletal function and cell adhesion as promising targets for identifying biomarkers and developing therapies, particularly for BD type I, which often presents a more severe course and prognosis (Delgado-Sequera et al. 2024; Grande et al. 2016).

One major obstacle in studying BD’s neurobiological underpinnings and identifying biomarkers is the challenge of obtaining neural cells directly from patients. However, advances in cellular models have provided valuable alternatives (Bellon 2024; Unterholzner et al. 2021). Models derived from living patients, such as neural cells from induced pluripotent stem cells (iPSCs) and olfactory neuroepithelium (ONE), have become indispensable tools (Bellon 2024; Unterholzner et al. 2021). These models offer unique insights into the dynamic neuronal processes involved in psychiatric disorders, from elucidating BD’s pathophysiology to exploring potential therapeutic interventions (Bellon 2024; Borgmann-Winter et al. 2015; Lavoie et al. 2017; Matigian et al. 2010; Perrottelli et al. 2024; Unterholzner et al. 2021). The ONE, which continuously generates sensory neurons throughout life, undergoes significant cytoskeletal reorganization during neurogenesis, making it a powerful model for studying BD and related disorders like schizophrenia (SZ) (Benítez-King et al. 2016). Research on cells derived from the ONE of BD patients has revealed notable findings. Alterations in microtubule organization—a key component of the cytoskeleton—have been observed, correlating with reduced migratory capacity (Muñoz-Estrada et al. 2015; Solís-Chagoyán et al. 2013). Additionally, increased levels of dihydropyrimidinase-related protein 1 (CRMP1), critical for neuronal cytoskeletal formation, were detected in these neurons, with stabilization occurring following lithium treatment (McLean et al. 2018). In this line, increased neuronal excitability in iPSC-derived hippocampal neurons from BD patients has been shown to be selectively reduced by lithium in neurons from patients who are clinical lithium responders (Mertens et al. 2015). Additionally, the number of CD34+ very small embryonic-like stem cells was similar to that in controls and negatively correlated with the duration of lithium treatment and serum lithium concentration, but only in lithium-treated patients (Ferensztajn-Rochowiak et al. 2017). These findings demonstrate that neurobiological mechanisms associated with BD and its pharmacological treatment are also reflected in stem cells.

In this study, we utilized ONE cells due to their multipotent progenitor population that naturally expresses neural markers (Durante et al. 2020). These cells offer a significant advantage as they require minimal manipulation and bypass the need for reprogramming, providing a non-invasive and rapid approach to obtaining neural cells (Unterholzner et al. 2021). By employing proteomics and RNA sequencing, we aimed to identify biomarkers and therapeutic targets related to the cytoskeleton and associated pathways, such as cell adhesion, movement, and morphology. Additionally, we investigated whether the identified neuroepithelial biomarkers could also be detected in blood samples, offering a more accessible option for clinical practice.

## Methods

### Participants

In total, 12 BD patients and 13 control subjects (CS), matched by age and gender, were recruited in the area of west Cádiz: Mental Health Clinical Management Unit of the Jerez—Costa Noroeste y Sierra de Cadiz. Health Management Area (Jerez University Hospital, Jerez, Spain) and Puerta del Mar Hospital Area (Cádiz, Spain), where the biological sample collection and clinical evaluation was also carried out. The Andalusian Ethics Committee (CCEIBA) approved this study, and all the participants signed the informed consent.

Inclusion criteria for participants were: (1) aged between 18–70 years; (2) for patients, diagnosis of BD type I according to DSM-5 (APA 2013) for CS, participants with no previous diagnosis of mental illness according to DSM-5. Exclusion criteria of all participants were: (1) the presence of organic diseases of the central nervous system; (2) history of head trauma with loss of consciousness; (3) other psychiatric disorders of axis I (for BD patients); (4) intellectual disability; (5) diagnosis of pervasive developmental disorders; (6) pregnancy and lactation; and (7) diseases affecting the olfactory mucosa (such as nasal polyps or sinusitis); presentation of symptoms related to respiratory or digestive pathologies, and SARS-CoV-2 infection at the time of sample collection. At the time of sample collection, all patients were receiving lithium and other psychotropic drugs. They were clinically stable, as confirmed by assessments with the HDRS, YMRS, and CGI scales (Table 1).

**Table 1:** Description of demographic and clinical characteristics

	Control	Bipolar disorder
Sex, *n*	7 M, 6W	9 M, 3W
Age, *y* (mean ± SD)	47.69 ± 12.15	49.75 ± 9.77
Illness, *y* (mean ± SD)	–	23.42 ± 7.57
Number of previous hospitalizations	–	3.42 ± 5.14
Number of previous episodes (mean ± SD)		
Depressive	–	8.92 ± 11.8
Manic	–	4.25 ± 5.63
Mixed	–	0.83 ± 1.4
Hypomanic	–	4.75 ± 7.12
Polarity, *n*		
No polarity	–	6
Depressive	–	5
Manic or hypomanic	–	1
HDRS (score ± SD)	3.23 ± 4.622	6.75 ± 6.538
YMRS (score ± SD)	0.54 ± 0.877	4.58 ± 3.988***
CGI (score ± SD)	1.08 ± 0.277	1.75 ± 0.754**
Medication, *n*		
Lithium	–	12
Valproic	–	4
Carbamazepina	–	1
Antidepressant	–	3
Antipsychotic	–	10
Benzodiazepines	–	5

*n*, number; *y*, years; SD, standard deviation; *s*, score; HDRS, Hamilton Depression Rating Scale; YMRS, Young Mania Rating Scale; CGI, Clinical Global Impression Scale; M, men; W, women. **p < 0.01; ***p < 0.001

### Clinical assessment

Psychiatric evaluation and assessment of DSM-5 criteria for BD type I were conducted using the Structured Clinical Interview for DSM-5 Disorders-Clinical Version (SCID-5-CV) (First et al. 2016). The Hamilton Depression Rating Scale (HDRS-21) (Hamilton 1960) was used to assess depressive symptoms, and the Young Mania Rating Scale (YMRS) was used for manic symptoms (Young et al. 1978). Total severity of illness was assessed using the Clinical Global Impression for Bipolar Disorder Modified (CGI-BP-M) scale (Spearing et al. 1997).

### Nasal brushing and cell culture

Cells were exfoliated from the nasal cavity as described previously (Delgado-Sequera et al. 2021; Galindo et al. 2018) to obtain samples from the middle and superior turbinates through a circular movement. Cells were grown at 37 °C with 5% CO_2_ in Dulbecco’s Modified Eagle Medium/Ham F-12 (DMEM/F12) containing 10% fetal bovine serum (FBS), 2% GlutaMAX 100X, and 0.2% primocin (Thermo Scientific, Spain). When confluent, the cells were detached with 0.25% trypsin–EDTA (GibcoBRL, USA), and approximately 200,000 cells were re-plated in 75 cm^2^ flasks and cultured in supplemented medium. Independent cell cultures were performed for each subject. All experiments were carried out on cells cultured to passage 6**.**

### Collection of human blood samples

Venous blood samples (10 mL) were collected in the morning after overnight fasting. Blood tubes were centrifuged at 1000*g* for 10 min at 4 °C, and the resulting plasma samples were stored at − 80 °C. PBMCs were then isolated by density gradient centrifugation (30 min at 900*g*; Ficoll® Paque Plus, GE Healtcare Life Sciences, Pittsburgh, PA, USA). The pellet was stored at − 80 °C until use.

### Immunocytochemistry

1 × 10^4^ cells were plated on 13-mm round glass coverslip and incubated at 37 °C in 5% CO_2_ prior to fixation for immunofluorescence. All assays were performed in triplicate. The cells were fixed with 4% paraformaldehyde (Sigma-Aldrich Chemicals, Spain) in PBS for 20 min and after washing in PBS, cells were kept for 1 h at room temperature in blocking solution containing 10% Bovine Serum Albumin (Sigma, Spain); 0.25% Triton-X-100; and 1% FBS in PBS. When anti-Ki67 was used, the cells were permeabilized with 0.2% Triton-X-100 in PBS for 25 min and then incubated for 20 min in blocking solution containing 4% FBS. The cells were then incubated overnight at 4 °C with the primary antibodies: mouse anti-β-III-Tubulin (1:1000 dilution; ThermoFisher (MA1-118), USA; anti-Ki67 (1:100 dilution; ThermoFisher, USA) in a buffer containing 0.03% Triton-X-100 and 3% BSA in PBS. The cells were incubated with the secondary antibodies for 1 h at room temperature: AlexaFluor488 donkey anti-Rabbit (1:1000 dilution; Invitrogen (A21206), USA) and AlexaFluor568 donkey anti-Mouse (1:1000 dilution; Invitrogen (A10037), USA). The nuclei were stained with 4′, 6′-diamidino-2-phenylindole dihydrochloride (DAPI, 1:5000 dilution; Panreac Applichem (A4099), Spain), and all the coverslips were then mounted with Fluoro-Gel medium (Electron Microscopy Sciences, USA). Images were acquired on an MMI CellCut plus (Olympus, Japan).

### Cell size and shape measurement

Neuronal microtubules were identified by β-III-tubulin immunostaining, as described above and in triplicate coverslips for each sample. A total of 100 cells were assessed per subject from 15 random microscopy fields. The area, perimeter and Feret’s diameter of these cells were defined and measured with the ImageJ software (USA).

### Wound healing assay

The assay was performed following the manual for the “Wound Healing Assay” (Ibidi, Germany). A 70 μl suspension of 3 × 10^5^ cells was added into each well of the 2-Well Culture-Insert and after 24 h, when the cell monolayer was confluent, the 2-Well Culture-Insert was removed and fresh medium was added. At that time (0 h) a wound was made in the culture, which was free of cells, and images were taken at 0 and 24 h, to quantify the number of cells migrating into the wound and the percentage of closure. Each sample was assayed in triplicate.

### Focal adhesion immunolabelling

To evaluate the cell focal adhesion, 2 × 10^3^ cells were plated on a coverslip as indicated in the commercial manual of the “actin cytoskeleton and focal adhesion staining kit” (Merck (FAK100), Spain). Cells were permeabilized with 0.5% Triton-X 100 in PBS for 30 min and then incubated in blocking solution containing 1% BSA for 1 h at room temperature. Cells were incubated with the primary antibody, mouse anti-vinculin (1:500 dilution: Merck (90227), Spain) at 4 °C overnight, and after three washes, the cells were incubated for 1 h at room temperature with TRITC-conjugated Phalloidin (1:1000, Merck (90228), Spain) and an AlexaFluor488 Donkey anti-Mouse secondary antibody (1:1000 dilution; ThermoFisher (A21202), USA). Images were acquired with an MMI CellCut plus (Olympus, Japan). To quantify focal points of adhesion immunolabelled with anti-vinculin, images were analyzed with ImageJ software (USA), and the total number of vinculin adhesion points were quantified per cell. Assays were performed in triplicate and a total of 10 cells per sample were analyzed.

### Proteomic studies

ONE cells were cultured under proliferative conditions for 48 h and then lysed (1 × 10^6^ cells per sample) in lysis buffer (1% NP40, 50 mM HEPES [pH 7], 150 mM NaCl, 1 mM EDTA) supplemented with protease inhibitors. In addition, the lysis of PBMCs obtained from patients (approximately 1 × 10^7^ cells) was also performed, and protein digestion was carried out as previously described (Delgado-Sequera et al. 2021). Briefly, proteins (100 μg/sample) from ONE cells and PBMCs were precipitated in acetone overnight at − 20 °C and recovered by centrifugation at 17,000*g* for 20 min at 4 °C. Protein pellets were resuspended in 8 M Urea in Tris 10 mM [pH 8], reduced with 10 mM dithiothreitol at 50 °C for 30 min, and alkylated with 50 mM iodoacetamide for 20 min at room temperature (RT) in the dark. Samples were digested for 4 h at RT with Lys-C enzyme/substrate (Promega (V167), USA) (enzyme/substrate ratio 1:50), and then diluted four times with 50 mM ammonium bicarbonate for further trypsin digestion (Promega, USA) at 37 °C overnight (enzyme/substrate ratio 1:50). The digested peptides were desalted using a SepPak C18 cartridge and dried in a SpeedVac, prior to mass spectrometry (MS) analysis using a label-free quantitative (LFQ) approach. Peptide samples (approximately 500 ng/sample) were loaded onto a nano-ACQUITY UPLC System (Waters, USA) using a 300 C18 UPLC trap column (180 μm × 20 mm, 5 μm: Waters), connected to a BEH130 C18 column (75 μm × 200 mm, 1.7 μm: Waters, USA). Peptides were eluted directly into an LTQ Orbitrap XL mass spectrometer (Thermo Finnigan, USA) at 300 nl/minutes and using a 120 min linear gradient of 3–50% acetonitrile. MS analysis was carried out in a data-dependent acquisition mode. Next, Progenesis LC–MS (Waters, USA) software was used for the LFQ analysis of differential protein expression. One run was used as a reference, to which the precursor masses in all the other samples were aligned. Only features comprising charges of 2+ and 3+ were selected, and the raw abundances of each feature were normalized automatically and converted to logarithms against the reference run. A peak list containing the information of all the features was generated and exported to the Mascot search engine (Matrix Science Ltd., UK). Differentially expressed proteins (DEPs) were defined as follows: p value (t-test) < 0.05 and fold-change rates > 1.5 for upregulated or < 0.6 for downregulated proteins identified in at least two replicates. Together with the fold change, the data were finally uploaded into the Ingenuity® Pathway Analysis (IPA) software (Ingenuity Systems, USA) and STRING (Szklarczyk et al. 2023) to investigate their molecular and biological functions.

### RNAseq studies

ONE cells were cultured under proliferative conditions for 48 h and subsequently frozen. RNA extraction was carried out using the miRNeasy Micro kit (QIAcube/Qiagen) according to the manufacturer's instructions. Total RNA library preparation was performed using the Illumina® Stranded Total RNA Prep kit, including ligation with Ribo-Zero Plus for ribosomal RNA depletion. Sequencing was conducted on the NovaSeq 6000 platform (Illumina, San Diego, CA, USA) using paired-end sequencing with a target depth of 60–100 million uniquely mapped reads per library. Quality control assessments were performed on raw sequencing data to ensure data integrity. Normalization of expression levels was carried out to adjust for sequencing depth and other technical variations. Differential expression analysis between the two experimental groups was conducted using the DESeq method, with False Discovery Rate (FDR) correction applied to account for multiple testing.

### Statistical analysis

In vitro statistical analyses were performed using IBM SPSS Statistics and GraphPad Prism software (GraphPad Software, USA). Normal distribution was evaluated with a Kolmogorov–Smirnov test. Data comparison between groups was assessed with unpaired Student’s *t* test (for normal distribution) or Mann–Whitney U test (for non-normal distribution). For qualitative variables, chi-squared test was used. The area under the Receiver Operating Characteristic (ROC) curve (AUROC) was utilized to identify potential discriminative biomarkers among the expressed proteins and RNAs in ONE cells, as well as the proteins in PBMCs. The AUROC quantifies the overall ability of the biomarker to discriminate between the two classes, with an AUROC of 1 representing a perfect classifier and an AUROC of 0.5 indicating no discriminative ability (random guessing).

The potential relationships between cellular parameters and molecules were assessed using correlation coefficients: Pearson’s for normally distributed data or Spearman’s for non-normal data. The differences were considered statistically significant at a p-value < 0.05.

## Results

### Sample characteristics

A total of 12 patients diagnosed with BD and 13 matched controls were recruited. There were no differences in sex (Chi-squared; p = 0.27) or age (Mann–Whitney U; p = 0.65) between the groups (Table 1). The patients had a minimum illness duration of 12 years, with an average duration of 23.42 years (± 7.57 SD; Table 1). Additional details on the course of illness, including previous hospitalization and episodes frequency and types of episodes, are also provided in Table 1. Regarding clinical characteristics, patients exhibited higher scores on both the YMRS and CGI scales (Mann–Whitney U; p < 0.001 and p = 0.007, respectively) compared to controls, with no differences on the HDRS scale (Mann–Whitney U; p = 0.076) (Table 1). In terms of medication, patients were receiving lithium and additional treatments such as anticonvulsants, antidepressants, antipsychotics or benzodiazepines (Table 1).

### Comparison of cellular characteristics between cells from bipolar disorder patients and controls

**Fig. 1:**
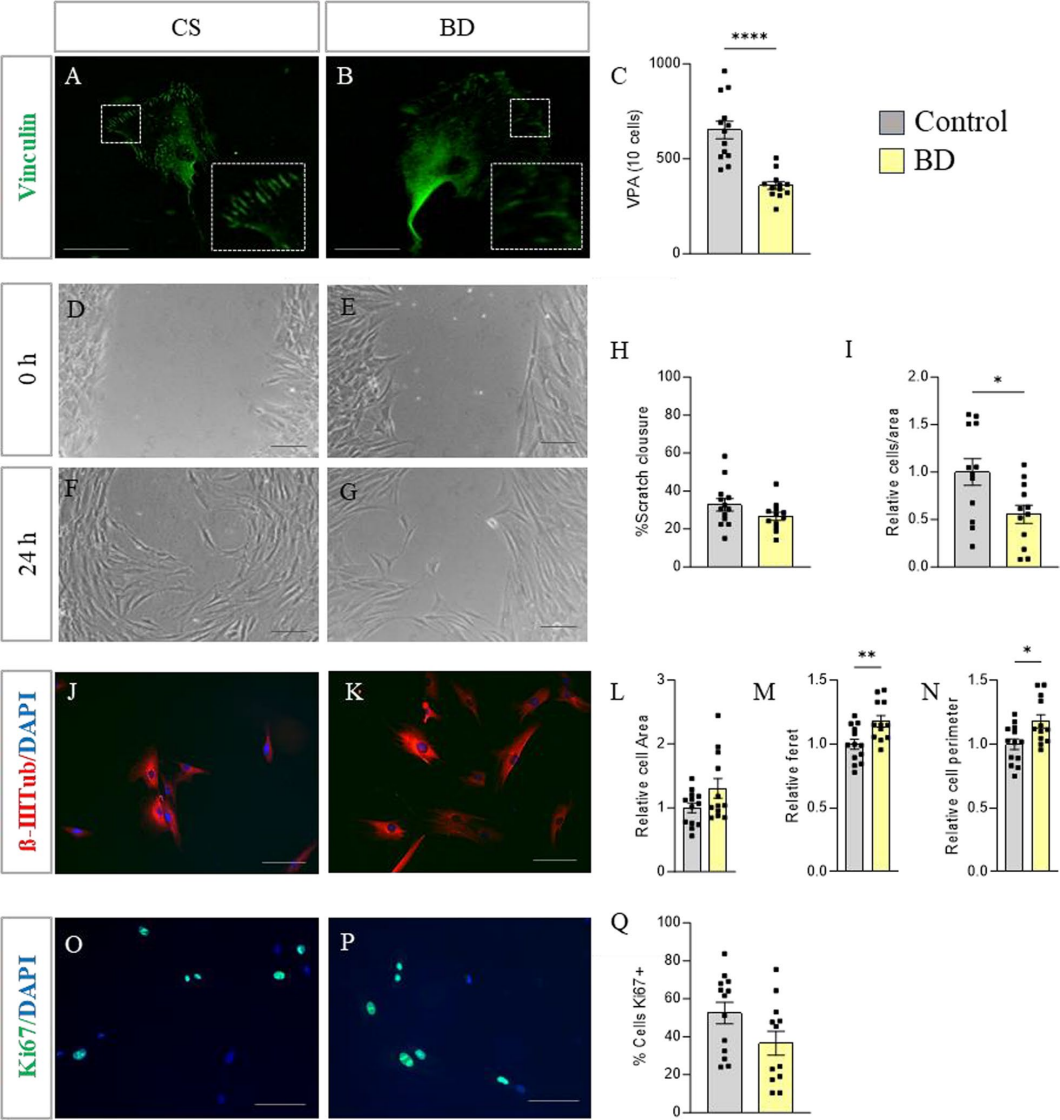
In vitro characterization of ONE cells. **A**, **B** Immunofluorescence images of vinculin-positive cells. The insets show magnifications of selected areas. **C** Quantification of vinculin points of adhesion (VPA). **D**–**G** Bright-field images of the scratched area at 0 and 24 h. **H** Quantification of the percentage of scratch closure after 24 h. **I** Quantification of the number of cells that migrated to the scratch after 24 h. **J**, **K** Immunofluorescence images showing β-III-Tubulin-positive cells (red) with nuclei stained with DAPI (blue). **L**–**N** Quantification of cell area, perimeter, and Feret’s diameter relative to that of the control cells. **O**, **P** Immunofluorescence images of Ki67-positive cells (green) with nuclei stained with DAPI (blue). **Q** Quantification of the percentage of Ki67-positive cells relative to the total number of cells quantified by DAPI. Data are presented as mean ± SEM. *p < 0,05; ** p < 0,01; **** p < 0,0001. Scale bar represents 50 μm. CS: Control subjects; BD: Bipolar disorder; VPA; vinculin points of adhesion

In vitro experiments with ONE cells were conducted to assess adhesion, migration capacity, and cell morphology as potential indicators of cytoskeletal alterations in cells from BD patients (Fig. 1A–N). In addition, the state of cell proliferation was also evaluated (Fig. 1O–K).

Thus, based on the number of vinculin adhesion points (Fig. 1A, B) a significant decrease in focal adhesions was observed in cells from BD patients compared to controls (Student’s *t*-test; p = 0.0001, Fig. 1C). Also, to evaluate cell migration, we conducted a ‘wound healing’ assay (Fig. 1D–G). Although no differences between groups were observed in wound closure 24 h post-scratch (Student’s t-test; p = 0.135, Fig. 1H), there was a decrease in the number of cells migrating to the wound area (Student’s t-test; p = 0.0152, Fig. 1I). Finally, cell size and morphology were assessed by quantifying the area occupied by microtubules labeled with β-III-tubulin antibody (Fig. 1J, K). Regarding cell area, no significant differences were observed between ONE cells from patients and controls (Student’s t-test; p = 0.072, Fig. 1L). However, a significant increase in the perimeter (Student’s t-test; p = 0.0133, Fig. 1M) and Feret’s diameter (Student’s t-test; p = 0.0062, Fig. 1N) was seen in BD derived cells compared to controls, suggesting morphological differences, likely indicating a larger and more elongated shape in patient cells.

Furthermore, to evaluate cell proliferation state, the number of cells expressing Ki67 in vitro was quantified (Fig. 1O, P). No significant differences were observed between cells derived from patients and controls (Student’s t-test; p = 0.0728, Fig. 1Q).

### Analysis of differentially expressed proteins and RNA

In order to identify molecular mechanisms altered in BD cells that might explain the functional and morphological changes described above, LFQ-proteomic and RNAseq analyses were conducted. Full information regarding protein/RNA identification and results derived from LFQ and RNAseq analysis can be found in Supplementary Tables 1, 4 and 7.

Further analysis with the Ingenuity Pathways Analysis software were carried out to investigate biofunctions, canonical pathways and disorders potentially associated with differentially expressed proteins (DEPs) and RNA (DEGs). This approach allowed us to identify significant patterns and associations that could shed light on the molecular underpinnings of BD, providing a deeper understanding of its pathophysiology.

**Fig. 2:**
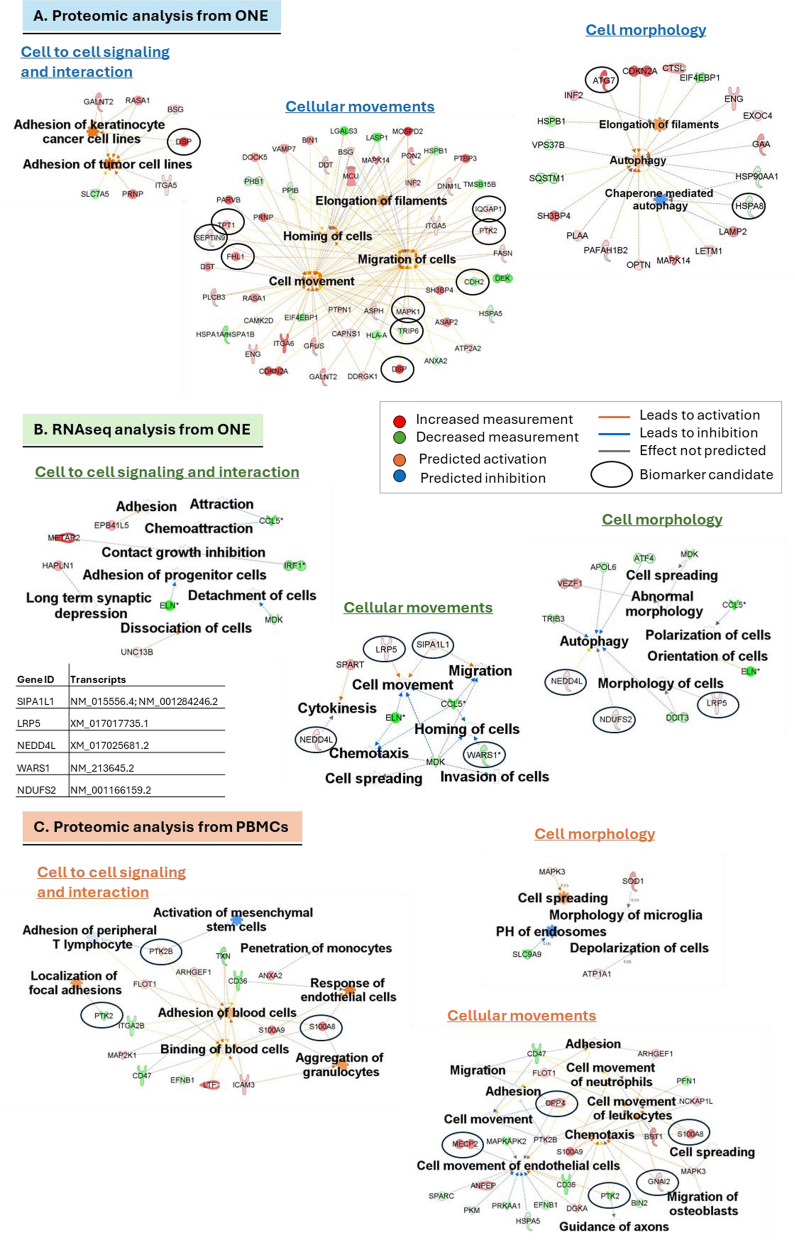
Biofunctions significantly affected in BD cells compared to controls: ‘cell to cell signaling and interaction’, ‘cell movement’ and ‘cell morphology’. **A** Selected biofunctions and proteins involved in these biofunctions from ONE cells. **B** Selected biofunctions and RNAs involved in these biofunctions from ONE cells. **C** Selected biofunctions and proteins involved in these biofunctions from PBMCs. *ONE* olfactory neuroepithelium, *PBMC* peripheral blood mononuclear cells. Circles label biomarker candidates with AUROC > 0.8 for proteins and AUROC = 1 for RNA transcripts

The results derived from both DEPs and DEGs, particularly in the context of in vitro assessments described above, reported a correlation between certain proteins related to ‘cell adhesion’ (‘cell to cell signaling and interactions’), ‘cell movement’ and ‘cell morphology’ (Fig. 2A, B; supplemental Tables 3, 6).

Remarkably, in agreement with the findings in ONE cells, the results derived from PBMCs also indicated an association between the DEPs with functions such as ‘cell-to-cell signaling and interactions’, ‘cellular movements’ and ‘cell morphology’, suggesting that the changes seen in neuroepithelial cells could also be detected in peripheral blood (Fig. 2C; supplemental Table 9).

**Fig. 3:**
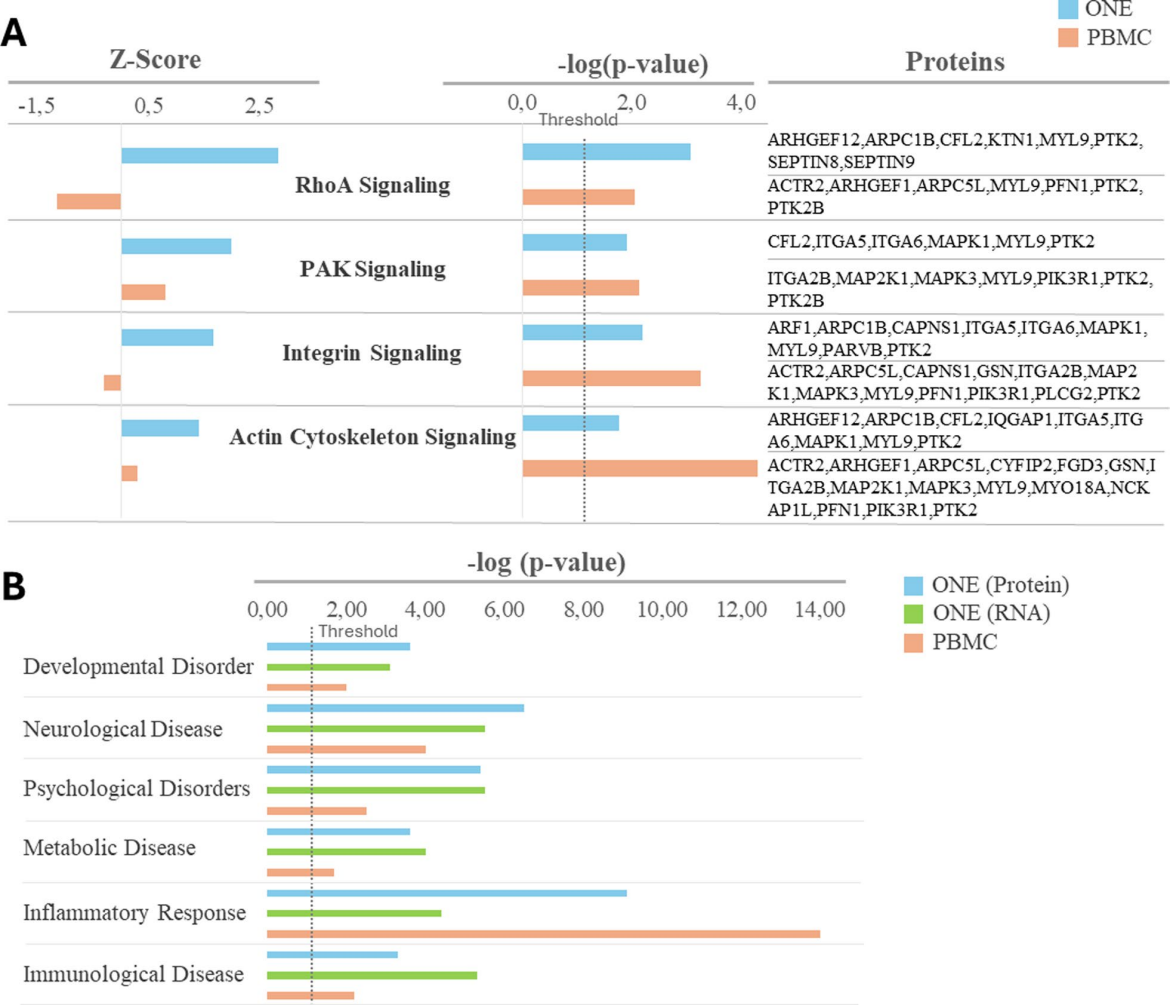
Cellular pathways and disorders associated with differentially expressed molecules in BD cells compared to controls. **A** Canonical pathways significantly altered in BD cells compared to controls and related to the cytoskeleton, cell migration and/or cell adhesion. **B** Disorders associated with the differentially expressed proteins in ONE and PBMC from BD patients compared to controls. *ONE* olfactory neuroepithelium cells, *PBMC* peripheral blood mononuclear cells, *PAK* p21-activated kinase

In addition, the proteins found to be altered in the ONE cells and PBMCs from BD individuals compared to controls, were significantly linked to pathways involved in cell adhesion, migration, and cytoskeletal dynamics, including ‘RhoA Signaling,’ ‘PAK Signaling,’ ‘Integrin Signaling,’ and ‘Actin Cytoskeleton Signaling’ (Fig. 3A; Supplemental Tables 2, 8). Notably, in ONE cells, these four pathways were predicted to be activated (z-score > 1) (Fig. 3A). Similarly, proteomics data from PBMCs revealed comparable pathway associations, with ‘PAK Signaling’ and ‘Actin Cytoskeleton Signaling’ also predicted to be activated, while ‘RhoA Signaling’ and ‘Integrin Signaling’ were predicted to be inhibited (z-score < 1) (Fig. 3A). However, these canonical pathways were not significantly affected in the RNA sequencing analysis (Supplemental Table 5). Overall, all these functions and pathways linked to proteins and RNAs differentially expressed in ONE cells, could potentially explain the observed decrease in adhesion capacity and migration, and with the different morphology observed in our in vitro assays (Fig. 1A–N).

Interestingly, the altered proteins and RNA from the ONE cells, as well as proteins from PBMCs, were associated to several disorders such as ‘developmental disorder’, ‘neurological disease’, ‘psychological disorders’, ‘metabolic disease’, ‘inflammatory response’, and ‘immunological disease’ among others (Fig. 3B; supplemental Tables 3, 6, 9).

### AUROC analysis

**Fig. 4:**
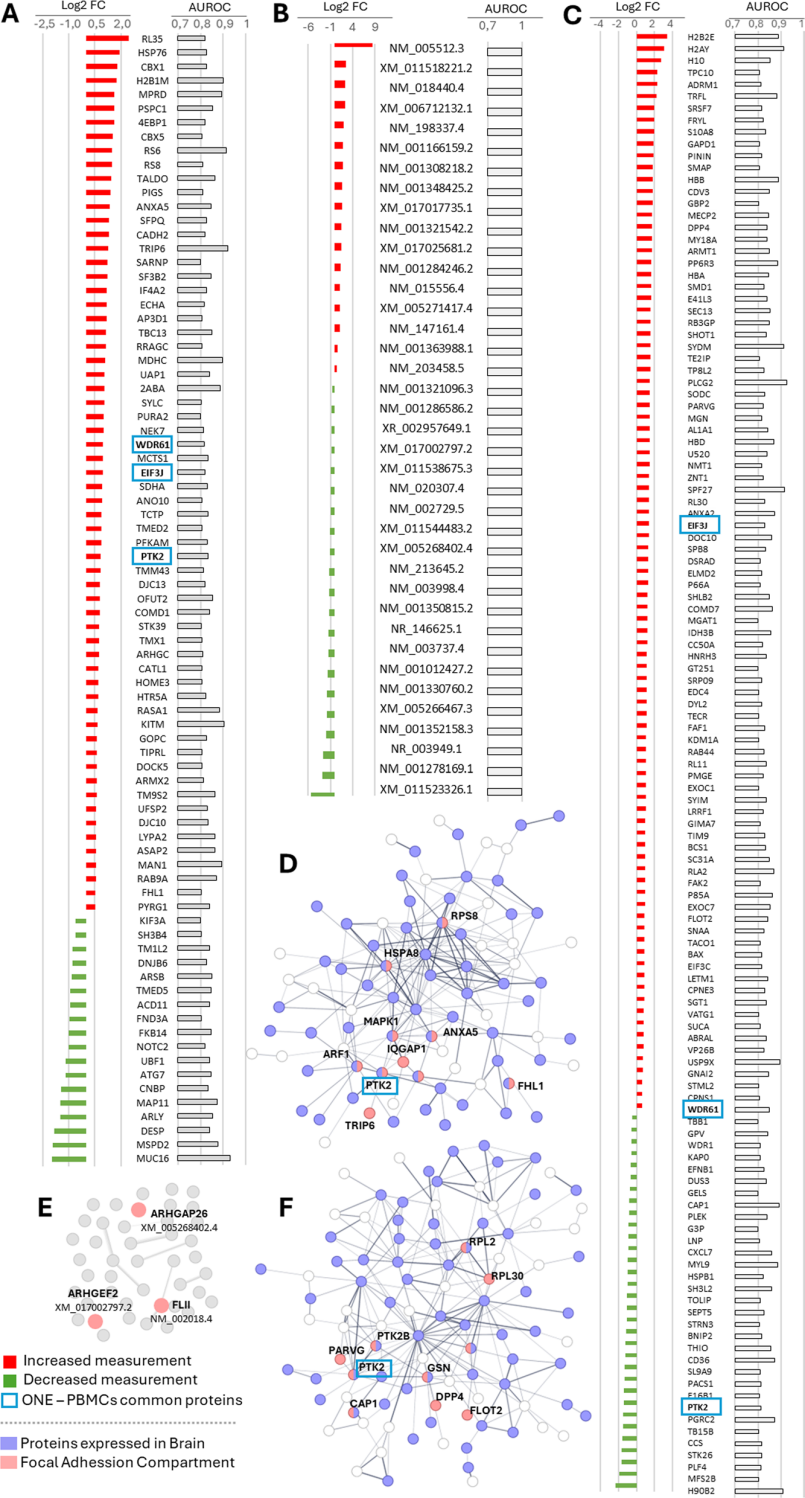
Proteins and RNA transcripts expression levels (BD vs Control) as biomarker candidates. **A** List of proteins from ONE with AUROC > 0.8 and Log2 Fold Change (FC) < − 0.5 or > 0.5. **B** List of RNA transcripts from ONE with AUROC = 1 and Log2 Fold Change (FC) < − 0.5 or > 0.5. **C** List of proteins from PBMC with AUROC > 0.8 and Log2 Fold Change (FC) < − 0.5 or > 0.5. **D** Proteins from ONE with AUROC > 0.8, including enrichment in proteins expressed in the brain (purple) and/or in the focal adhesion compartment (pink), as assessed by STRING. **E** RNA transcripts from ONE with AUROC = 1; including RNA transcripts with a function in focal adhesion (pink). **F** Proteins from PBMC with AUROC > 0.8, including enrichment in proteins expressed in the brain (purple) and/or in the focal adhesion compartment (pink), as assessed by STRING

To identify potential biomarkers in BD, proteins or RNA transcripts were assessed by measuring the AUROC, considering molecules with an AUROC greater than 0.8 as good candidates. Thus, from the ONE sample, 111 proteins and 3034 RNA transcripts (46 of which demonstrated an AUROC = 1) could be potential biomarker candidates. From these molecules, 81 proteins and 38 RNA transcripts were selected as they presented a log2 fold change < − 0.5 or > 0.5 (Fig. 4A, B; supplemental Table 10). In PBMC samples, 128 proteins were selected, with 123 showing a log2 fold change < − 0.5 or > 0.5 (Fig. 4C; supplemental Table 10). Among these, 3 proteins overlapped with those identified in the ONE samples: WD repeat-containing protein 61 (WDR61, log2 fold change 0.62 for ONE protein and 0.58 for PBMC protein), Eukaryotic translation initiation factor 3 subunit J (EIF3J, log2 fold change 0.65 for ONE protein and 1.3 for PBMC protein), and focal adhesion kinase 1 (FAK1 or PTK2, log2 fold change 0.75 for ONE protein and − 1.43 for PBMC protein) (Fig. 4A, C; supplemental Table 10). Given our focus on proteins with an AUROC greater than 0.8, we concentrated on those expressed in brain tissue, particularly those showing enrichment in focal adhesion pathways identified by STRING (Fig. 4D, F; images sourced from STRING).

**Fig. 5:**
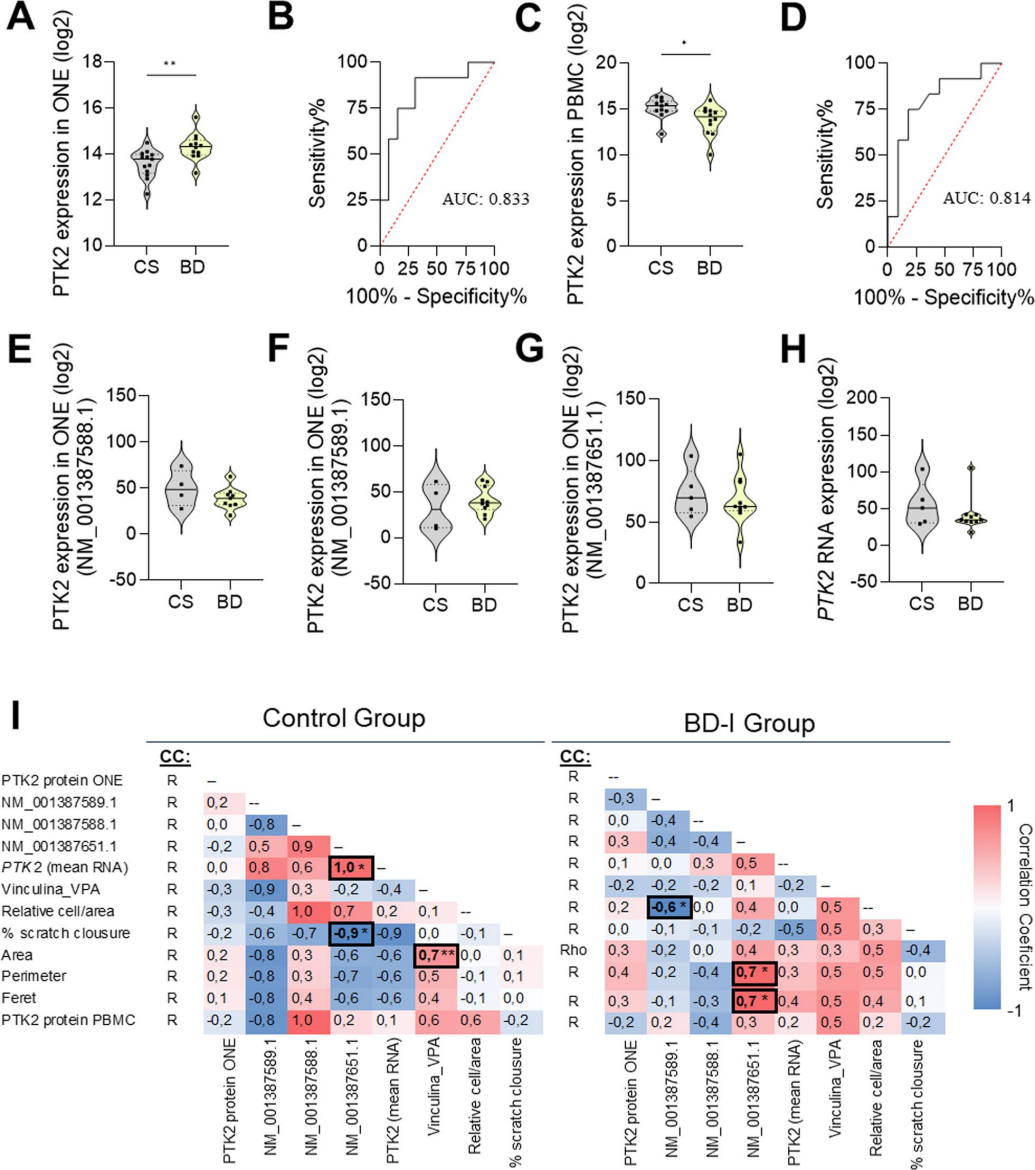
Measurement of PTK2 expression levels and correlations with cellular parameters. **A**, **B** PTK2 protein expression levels and AUROC in ONE. **C**, **D** PTK2 protein expression levels and AUROC in PBMC. **E**–**H** *PTK2* RNA transcript levels in ONE. **I** Correlation coefficient between PTK2 protein/RNA expression levels and cellular parameters. *CS* control subjects, *BD* bipolar disorder, *ONE* olfactory neuroepithelial cells, *PBMC* peripheral blood mononuclear cells; *p < 0.05; **p < 0.01; R: Pearson’s correlation; Rho: Spearman’s correlation

STRING analysis with selected RNA transcripts (AUROC = 1) did not show enrichment in the focal adhesion compartment, however, 3 of these transcripts were involved in the focal adhesion function: XM_005268402.4 [Rho GTPase activating protein 26 (ARHGAP26), transcript variant X21], XM_017002797.2 [Rho/Rac guanine nucleotide exchange factor 2 (ARHGEF2), transcript variant X13] and NM_002018.4 [FLII actin remodeling protein (FLII), transcript variant 1] (Fig. 4E). From the proteins enriched in focal adhesion compartment, only PTK2 was present in both ONE and PBMC lists with AUROC > 0.8 (Fig. 5B, C). This protein showed a significantly increased expression in ONE cells from BD compared to controls (Student’s t-test; p = 0.0051, Fig. 5A) but a decrease in PBMC (Student’s t-test; p = 0.0319, Fig. 5C). RNAseq analysis identified three RNA transcripts from the *PTK2* gene (NM_001387589.1, NM_001387588, NM_001387651); however, no differences were found between the BD and control groups, either in the individual transcripts or in the mean expression levels of these transcripts (Fig. 5E–H).

Next, we examined the correlations between PTK2 protein expression levels in ONE and PBMC, as well as RNA transcripts from ONE, with cellular parameters such as vinculin adhesion points, cell migration, and cell size/shape (Fig. 5I). In the BD group, the PTK2 mRNA transcript “NM_001387589.1” exhibited a significant negative correlation with the number of migrating cells, indicating that higher levels of this transcript were associated with reduced cell migration in the BD group. Additionally, the PTK2 mRNA transcript “NM_001387651.1” showed a significant positive correlation with cell perimeter and Feret’s diameter, suggesting that higher levels of this transcript are linked to larger and more elongated shape. In contrast, the control group did not show any such correlations (Fig. 5I).

Furthermore, no correlation was found between PTK2 protein levels in ONE and PBMC, nor with PTK2 transcript levels in ONE (Fig. 5I).

## Discussion

The goal of this study was to identify specific cellular and molecular alterations that could serve as biomarkers and potential therapeutic targets in patients with BD. We found that ONE cells derived from BD patients exhibited decreased substrate adhesion, reduced cell migration, and morphological changes compared to control cells. Through proteomic and RNAseq analysis, we identified the molecular mechanisms that may underlie these alterations. Our findings provide deeper insights into the pathophysiology of BD and pave the way for the identification of diagnostic biomarkers and the development of more targeted and effective treatments.

The neurodevelopmental hypothesis of BD suggests that alterations in neuronal migration and synaptogenesis during prenatal stages lead to abnormal brain maturation (Benítez-King et al. 2016; O’Shea and McInnis 2016). Consistent with this, cytoarchitectural abnormalities have been observed in postmortem brains of BD patients (Gigase et al. 2019), and cytoskeletal dysfunction has been implicated based on proteomic analyses of the human prefrontal cortex (English et al. 2009). Previous studies on ONE cells derived from BD patients have shown cytoskeletal alterations; labeling microtubules with β-III-tubulin revealed that the unstained area relative to the total cell area was significantly larger in BD patients compared to controls (Solís-Chagoyán et al. 2013), suggesting a more fragmented microtubular pattern in BD ONE cells. Our study further demonstrates that BD ONE cells exhibit distinct morphological alterations, including a larger and more elongated shape. Additionally, BD ONE cells demonstrated a reduced migratory capacity through a Transwell membrane within 6 h (Muñoz-Estrada et al. 2015). In contrast, our study found no significant differences between groups in wound closure 24 h post-scratch, suggesting that disparities in migration speed are more evident at earlier time points. However, we observed a decrease in the number of cells migrating to the wound area, which could be attributed to the morphological differences in BD cells. These structural changes may also contribute to the reduced number of vinculin adhesion points in BD cells compared to controls, indicating decreased adhesion capacity. Supporting this, previous studies have identified single-nucleotide polymorphisms in genes related to the cell adhesion molecule pathway in BD patients (O’Dushlaine et al. 2011), a pathway crucial for cell signaling and synaptic formation. Interestingly, ONE cells from BD patients did not show proliferation alterations, contrasting with findings from iPSCs derived from BD patients (Delgado-Sequera et al. 2024). Collectively, our findings, alongside previous research, highlight that BD ONE cells exhibit alterations in microtubule organization, cell migration, and adhesion capacity, which may impact brain maturation and function from early neurodevelopmental stages.

To go deeper into the molecular and cellular pathways behind these cellular alterations, we performed proteomic and RNAseq analysis on ONE cells derived from BD patients and controls. Consistent with our in vitro assay results, certain biofunctions, such as ‘cell-to-cell signaling and interaction,’ ‘cellular movement,’ and ‘cell morphology,’ were predicted to be altered. Notably, these biofunctions were also affected in BD PBMCs, highlighting their potential as easily accessible biomarkers for psychiatric disorders, given the routine use of blood samples in clinical practice (García-Gutiérrez et al. 2020; The Lancet Psychiatry 2016). Additionally, we identified canonical pathways that could contribute to the observed cellular alterations, primarily those related to cytoskeletal function. Key pathways, including RhoA/PAK/Integrin and Actin Cytoskeleton Signaling, were predicted to be activated in ONE cells from BD patients compared to controls, though they showed reduced or even decreased activation levels in PBMCs. This differential pathway activation underscores the complexity of cytoskeletal dysfunction in BD and highlights potential targets for further exploration. The RhoA signaling pathway is crucial for the organization of the actin cytoskeleton and plays a significant role in regulating dendritic spine density and length in pyramidal neurons. Consequently, changes in the expression of genes related to this pathway could lead to alterations in neuronal connectivity (Kermath et al. 2020). Consistent with our findings, previous research has reported overactivation of the RhoA signaling pathway in the prefrontal cortex of BD patients (Kermath et al. 2020). Additionally, several studies have demonstrated that increased expression or activity of RhoA reduces dendritic length and density, which are alterations observed in the prefrontal cortex of BD patients (Kermath et al. 2020; Konopaske et al. 2014). PAK signaling also plays a role in actin cytoskeleton dynamics and has been implicated in cell signaling, neuronal pathophysiology, cell morphogenesis, and survival (Crawford et al. 2012; Dobrigna et al. 2023). Differential expression of PAK genes has been identified in patients with SZ, BD, and other psychiatric disorders, suggesting its broader relevance. Notably, PAK inhibitors have been proposed as potential therapeutic approaches for brain disorders (Crawford et al. 2012; Dobrigna et al. 2023). Given our findings, which predict PAK signaling activation in BD ONE cells, further studies using PAK inhibitors would be valuable to determine whether they can restore normal morphogenesis in BD cells. Regarding integrins, these membrane proteins are expressed in most cells, mediating adhesion to other cells or to the extracellular matrix (Cui et al. 2024). Integrin activity in the nervous system plays an important role in regulating neurite outgrowth and synaptic plasticity, processes required for neuronal connectivity that are indeed affected in neurodevelopmental disorders (Lilja and Ivaska 2018). In our ONE samples from BD patients, we observed that integrin signaling was expected to be activated, whereas it was inhibited in the PBMCs. This indicates that different proteins and/or levels of expression are mediating this pathway in the different cell types. Consequently, the involvement of these proteins in the pathway does not necessarily imply identical expression profiles, as they may drive different actions depending on the specific cell type. This finding could be particularly interesting for further studies on the role of integrins in neural tissue, which remains to be fully understood, especially in the context of neurodevelopmental disorders (Lilja and Ivaska 2018). As mentioned, the pathways discussed above require cytoskeleton reorganization. Indeed, the actin cytoskeleton is one of the signaling pathways predicted to be activated in both ONE and PBMCs derived from BD patients. Actin cytoskeleton organization is essential for functions such as cell adhesion, migration, neuronal outgrowth, and synapse formation and maintenance (Zhao et al. 2015). In agreement with our study, previous research on postmortem samples from the cingulate cortex and hippocampus of BD and SZ patients has described alterations in this signaling pathway (Zhao et al. 2015).

Our proteomic and RNAseq analysis identified pathways and disorders associated with DEGs and DEPs in samples from patients with BD compared with controls. The most significant pathways identified in both ONE cells and PBMCs were the ‘inflammatory response’ and ‘immunological diseases’. These findings align with existing literature, indicating that neuroinflammation and immune dysfunction are present in at least a subset of BD patients (Delgado-Sequera et al. 2024; Mazza et al. 2018; Rosenblat et al. 2015). Although the causal relationship between BD development and immune-inflammatory processes remains unclear, accumulating evidence suggests that immune-inflammatory alterations are not merely secondary phenomena. For instance, increased incidences of immune-inflammatory events during early life stages of BD, along with the bidirectional relationship between BD and autoimmune disorders, have been reported (Khandaker et al. 2017; Misiak et al. 2018). This association is also observed in first-degree relatives, suggesting a heritable component (Sayana et al. 2017). Altered RNA transcripts in unaffected relatives of individuals with mood disorders further suggest that immune system disruptions may represent a trait marker of psychiatric vulnerability (Barnett and Smoller 2009). Dysregulation in inflammation-controlling mechanisms has been documented in BD across both chronic and early stages (Poletti et al. 2024). Importantly, ongoing efforts aim to correlate immune-inflammatory changes with potential biomarkers for diagnosis, prognosis, and treatment response in BD (Poletti et al. 2024; Ruiz-Sastre et al. 2024), similar to approaches being developed for other psychiatric conditions like schizophrenia (Leza et al. 2015). Accordingly, it has been shown that the mRNA expression of glial markers (Olig1 and Olig 2) examined in peripheral blood, was higher in BD patients not taking lithium than in the age- and sex-matched, healthy control subjects. Interestingly, in lithium-treated BD patients, GFAP and Olig1 expression levels were comparable to those in the control group, suggesting that lithium’s effect on mRNA glial markers may result from a reduction in the inflammatory response in the central nervous system (Ferensztajn-Rochowiak et al. 2016).

To identify potential diagnostic biomarkers, we conducted AUROC analysis on identified proteins and RNA transcripts. Proteomic analysis of both ONE and PBMC samples identified WDR61, EIF3J, and PTK2 as DEPs between BD cells and controls. Notably, WDR61 and EIF3J showed increased expression in both ONE and PBMC samples. WDR61 is involved in transcriptional regulation and downstream events of RNA synthesis, such as RNA surveillance (Zhu et al. 2005). Previous studies have demonstrated significant upregulation of WDR61 in peripheral blood from patients with first-episode psychosis (Leirer et al. 2019). EIF3J is a eukaryotic initiation factor (eIF) that plays a key role in stabilizing ribosomal preinitiation complexes at the start codon, functioning as an essential element of post-transcriptional gene regulation. Specifically, EIF3J, part of the translation initiation factor 3 complex, is located in the decoding center of the human 40S ribosomal subunit, regulating access to the mRNA-binding cleft in response to initiation factor binding (Fraser et al. 2007). This aligns with previous findings of altered expression in genes related to protein biosynthesis in biopsied ONE from BD patients (McCurdy et al. 2006). Additionally, EIF3J has been implicated in the development of positive symptoms in schizophrenia, potentially through regulatory mechanisms in peripheral blood (Jin et al. 2024). Genome-wide association studies from the Psychiatric Genetics Consortium and analysis of ONE cells from SCZ patients also show alterations in the EIF pathway (English et al. 2015), underscoring the role of EIF3J in psychiatric disorders.

Another identified protein, PTK2, was upregulated in ONE cells from BD patients compared to controls. PTK2, upon autophosphorylation, promotes membrane protrusion and adhesion turnover, which are critical processes in actin cytoskeleton dynamics. In neurons, these functions are vital for axon guidance and neural plasticity, with PTK2 colocalizing with actin at growth tips to facilitate movement (Armendáriz et al. 2014). While *PTK2* transcript levels (NM_001387589.1, NM_001387588.1, NM_001387651.1) did not significantly differ between BD patients and controls, increased *PTK2* RNA expression was associated with reduced cell perimeter and Feret’s diameter, along with a higher number of migrating cells in scratch assays (relative cell/area). These morphological traits distinguish BD ONE cells from control cells. In contrast, PTK2 was downregulated in PBMCs from BD patients compared to controls, suggesting that PTK2’s role diverges significantly between the central nervous system and the periphery due to distinct regulatory mechanisms, environments, and functional requirements. This pattern reflects similar disparities observed with other molecules that exhibit different expression profiles across these systems. Therefore, AUROC analysis identified WDR61, EIF3J, and PTK2 as potential biomarkers, showing altered expression in BD cells and PBMCs, suggesting distinct roles.

Regarding RNA analysis, no overlaps were found between RNAs and proteins with an AUROC > 0.8. This is understandable because RNA level quantification is not directly linked to the absolute protein levels it encodes, and it does not account for post-translational modifications. Therefore, RNA analyses offer predictions based on sequence data, detecting the presence of genomic transcripts that will encode the protein. However, three potential biomarker candidates encoding proteins related to focal adhesion were identified: ARHGAP26, ARHGEF2, and FLII. Notably, these RNAs have not been previously linked to BD in the literature (Konopaske et al. 2014). However, other members of the ARHG family are involved in canonical pathways, such as RhoA signaling and actin cytoskeleton signaling, which were significantly altered in ONE cells and PBMCs from BD patients, suggesting further exploration is warranted.

Our study has several limitations. First, the relatively small sample size suggests the need for larger cohorts to validate our findings. This limited sample size also prevented us from conducting more detailed analyses, such as examining the impact of sex or other clinical and demographic variables, which could offer additional insights into our results. Second, all patients in our study were undergoing treatment with lithium and other psychotropic drugs; for example, 10 out of 12 patients were also on antipsychotic medication. Therefore, we cannot rule out the influence of these drugs on the observed effects. This is particularly important in the case of lithium, as extensive literature suggests that patients who respond exceptionally well to lithium are neurobiologically distinct from the broader bipolar population (Vieta et al. 2018). Future studies involving treatment-naïve patients will be crucial to evaluate the potential of diagnostic biomarkers without the confounding effects of medication. Additionally, one strategy for addressing the potential medication confound in future experiments could involve incubating ONE cells from CS controls chronically in culture with the drug in question. This would help determine if the drug contributes to the cellular and molecular disturbances observed in BD cell lines. Moreover, the differential proteomic and expression patterns identified in our study may be related to the varying effects of lithium (or other psychotropic drugs) on ONE cells compared to PBMCs, warranting further investigation.

## Conclusions

In summary, our study on ONE cells from BD patients revealed cytoskeletal alterations, reduced adhesion capacity, and disrupted migration patterns. Proteomic and RNAseq analyses identified significant changes in pathways such as RhoA/PAK/Integrin and Actin Cytoskeleton Signaling, which contribute to these cellular abnormalities. Additionally, prominent immune-inflammatory responses highlight their potential as biomarkers. AUROC analysis identified proteins like PTK2 as potential diagnostic biomarkers, showing altered expression in both ONE cells and PBMCs. Notably, PTK2 RNA expression correlates with distinct morphological traits in BD ONE cells compared to controls, further emphasizing its research potential.

## Supplementary Information

Supplementary Material 1.

## Data Availability

Additional datasets from this study are available from the corresponding author upon reasonable request.

## Abbreviations

ARHGAP26: Rho GTPase activating protein 26
ARHGEF2: Rho/Rac guanine nucleotide exchange factor 2
AUROC: Receiver Operating Characteristic (ROC) curve
BD: Bipolar disorder
CGI-BP-M: Clinical Global Impression for Bipolar Disorder Modified scale
CRMP1: Dihydropyrimidinase-related protein 1
CS: Control subjects
DAPI: With 4′, 6′-diamidino-2-phenylindole dihydrochloride
DEGs: Differentially expressed RNA
DEPs: Differentially expressed proteins
DMEM/F12: Dulbecco’s Modified Eagle Medium/Ham F-12
eIF: A eukaryotic initiation factor
FAK1 or PTK2: Focal adhesion kinase 1
FBS: Fetal bovine serum
FDR: False Discovery Rate
FLII: Actin remodeling protein
HDRS-21: Hamilton Depression Rating Scale
iPSCs: Induced pluripotent stem cells
IPA: The Ingenuity® Pathway Analysis
LFQ: Label-free quantitative
MS: Mass spectrometry
ONE: Olfactory neuroepithelium
PBMCs: Peripheral blood mononuclear cells
RT: Room temperature
SCID-5-CV: Clinical Version
SZ: Schizophrenia
WDR61: WD repeat-containing protein 61
YMRS: Young Mania Rating Scale
